# The effects of price and non-price policies on cigarette consumption in South Africa

**DOI:** 10.18332/tid/123424

**Published:** 2020-07-23

**Authors:** Ernest N. Tingum, Alfred K. Mukong, Noreen Mdege

**Affiliations:** 1 Department of Economics, University of Namibia, Windhoek, Namibia; 2 Research Unit on the Economics of Excisable Products, School of Economics, University of Cape Town, Cape Town, South Africa; 3 Department of Health Sciences, University of York, York, United Kingdom

**Keywords:** price, non-price, tobacco policy, cigarette consumption, South Africa

## Abstract

**INTRODUCTION:**

The health consequences of smoking are serious and have been frequently detailed. A reduction in tobacco-related mortality hinges upon the ability to reduce tobacco usage. There is overwhelming evidence that higher cigarette prices reduce the demand for cigarettes, but little is known about the combined effect of price and non-price policies. This paper seeks to extend the analysis of price elasticities by estimating the combined effect of changes in price and non-price legislations in South Africa.

**METHODS:**

Annual time-series data from 1961 to 2016 are used, with a policy index constructed to capture the instances of non-price tobacco legislation. We estimate the combined impact of changes in tobacco control policy on cigarette consumption using a vector error correction model (VECM) and a two-stage least squares (2SLS) model.

**RESULTS:**

The estimated long-run own-price elasticities lie between -0.55 and -0.72, while the income elasticities lie between 0.39 and 0.49. The coefficients of the changing tobacco control policies and the changing market structure show that they contribute to a modest reduction in cigarette consumption. The short-run deviations from the steady state are presented using the error correction term (ECT).

**CONCLUSIONS:**

Cigarette demand is responsive to cigarette prices and non-pricing policies but failure to control for non-pricing policies overstates the price effect. This suggests that both cigarette prices and non-pricing legislation are effective in reducing cigarette consumption.

## INTRODUCTION

Smoking is known to have serious and welldocumented health consequences. As a result of the elevated risk of smoking-related diseases, a person who took up smoking early in life can be expected to die about 6 years earlier than a comparable nonsmoker^1^. According to Statistics South Africa (2017), 19.5% of the total deaths reported in 2016 were smoking-related, which accounts for approximately 81975 deaths. The ability to reduce the number of tobacco-related deaths, therefore, hinges on the ability to reduce tobacco consumption. Tobacco taxes and a number of tobacco-control policies have been implemented around the world with the objective of increasing the cost of purchasing tobacco products and reducing tobacco consumption.

Previous evidence has shown that increased cigarette prices are one of the most effective tobaccocontrol strategies in reducing tobacco consumption^2^. On the other hand, relatively little attention has been given to the effect of non-pricing tobacco legislation. The literature on the elasticity of demand for cigarettes can be classified into two strands. The first strand, quite sizable, completely ignores the role of nonpricing policies when estimating the price elasticity of demand for cigarettes^2^. The other strand accounts for the effect of other tobacco control legislations^3^. In this paper, we argue that there is a need for a simultaneous evaluation of pricing and non-pricing tobacco-control policies in order to reduce the bias associated with the price elasticity of demand for cigarettes. The price elasticity of demand for tobacco products will be overstated if there is failure to control for non-pricing policies.

The few studies that have considered the role of non-pricing policies simply introduced a dummy variable into the cigarette demand function^4-6^. The use of a dummy variable may not adequately address this problem, especially for economies that frequently amend their tobacco control legislation. Joossens and Raw^7,8^ provided weights for the different tobacco control policies that can be used for constructing a tobacco-control policy index. This measure is particularly important when estimating the effect of non-pricing laws for a country that has systematically amended its tobacco-control legislation. Few studies have used a comprehensive measure of other pieces of legislation when estimating the price elasticity of demand for cigarettes^1,9^. This paper contributes to the literature by simultaneously estimating the effect of price and non-pricing policies on tobacco consumption in South Africa, a developing country that has consistently amended its tobacco-control laws in order to significantly reduce the level of adult cigarette consumption.

Compared to many low- and middle-income countries (LMICs), South Africa is noted for using heavy excise taxes and other tobacco-control policies to reduce per adult cigarette consumption by almost half within 15 years^10,11^. For example, adult smoking prevalence decreased from a third of the adult population to about a fifth between 1994 and 2012^12^. While this decline is attributed mainly to the increase in excise taxes, the influence of numerous non-pricing policies, including banning tobacco advertising and sponsorship, as well as banning smoking in public and work places, cannot be ignored. In addition, smoking prevalence is still significantly high among adults^13^. Recent evidence from individual-level panel data indicates that the conditional price elasticity of demand for cigarettes in South Africa decreased from -0.305 to -0.303, after controlling for non-pricing policies^14^. However, this study, like previous studies in South Africa^5,6^, used a dummy to capture the effect of non-pricing policies. Other empirical studies have analyzed the relationship between real cigarette prices and the demand for cigarettes in South Africa, ignoring the effects of non-pricing tobacco control legislation^6,12,15-20^. The current study differs from these studies in that it considers more comprehensive measures of non-pricing policies.

This study addresses the evidence gap by estimating the combined impact on consumption of price changes, changes in legislation, and changing market structure where the tobacco industry experienced a transition from a near monopoly to a more competitive market structure^13^. We show that ignoring antismoking legislation overstates the price effects but understates the income effects both in the long-term and the short-term. Our results contribute to the literature on the effect of tobacco-control policies on cigarette consumption. A better understanding of this relationship can help inform the discussion on appropriate policies that will further reduce tobacco use. The study employs the econometric techniques of the Augmented Dickey-Fuller (ADF) and Phillip-Perron (PP) unit root tests, the Johansen cointegration test, the vector error correction model (VECM) and a two-stage least squares (2SLS) estimation in the analysis.

## METHODS

### Theoretical model

This paper uses the demand model to estimate the effect of price and non-price tobacco control legislation on cigarette consumption in South Africa from 1961 to 2016. There is a well-established relationship between price and income and cigarette consumption. The demand function for cigarettes is expressed as follows^9,16,21^:

Q_t_=f(P_t_,Y_t_,A_t_,D_t_)

where *Q_t_* is the per capita cigarette consumption in period *t*, *P_t_* is the price of cigarettes which has been adjusted for inflation (2016=100), *Y_t_* is the real per capita Gross Domestic Product (GDP), *A_t_* is the index of the non-price tobacco control policies, and *D_t_* is the dummy variable for the change in market structure that occurred in 2010. The short-run and long-run analyses in this study are based on the demand function given above.

### Data sources

**Figure 1:**
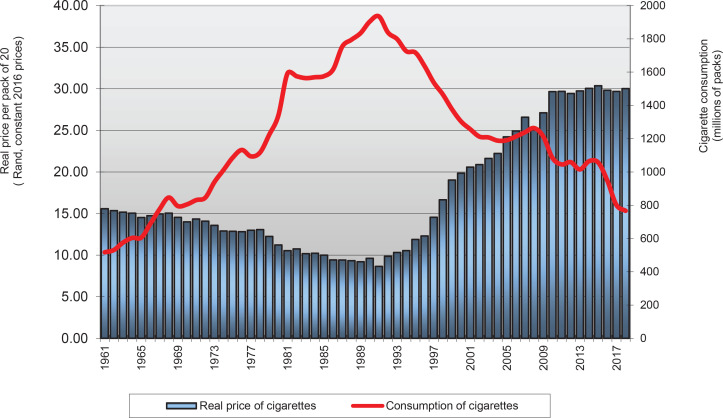
Real prices per pack and aggregate cigarette consumption, South Africa, 1961–2017

The analysis uses annual time-series data for the period 1961 to 2016. The data are extracted by DataFirst from Statistics South Africa (StatSA)^22^ and South African Reserve Bank reports. DataFirst (based at the University of Cape Town) provides open access to data from South Africa and other African countries by compiling and frequently updating information based on reports (https://www.datafirst.uct.ac.za/ dataportal/index.php/catalog/StatsSA/about). For this study, the dependent variable is annual adult percapita cigarette consumption. This is calculated by dividing the aggregate consumption by the size of the adult population (aged ≥15 years). The relationship between aggregate cigarette consumption and real cigarette prices in South Africa is presented in Figure 1. This figure shows substantial increases in the real price of cigarettes since 1994 as a result of the implementation of an aggressive excise tax policy. In contrast to the period before 1994, when real prices were falling, the average real price per pack increased by 190% between 1994 and 2012 and has remain almost constant afterwards^11^. Cigarette consumption increased during the period 1961–1993, started falling moderately, then its fall accelerated from 1995 through 2000. The decrease can be attributed to the policies, adopted by the government in 1994, that reduced smoking prevalence. The decrease in smoking prevalence is also attributed to the excise tax increment of 25% in 1994, 25% in 1995 and 18% in 1996^23^. In 1997, the government announced a 52% increase in the excise tax on cigarettes, which was expected to bring the total tax burden to 50% of the average retail selling price^5,11^. The total tax burden was revised to 52% of the average selling price in 2004^24^. As shown in Figure 1, the decline in cigarette consumption and smoking prevalence from about a third to less than a fifth between 1994 and 2012 is partly attributed to the substantial increase in real cigarette prices^12,14^. From 2013 onward, the steady decline in aggregate cigarette consumption is attributed to the effectiveness of price and non-price policies.

The independent variables include real prices (adjusted for inflation by dividing the nominal prices by the consumer price index, using 2016 as the base year), real per capita GDP, a policy index, and a dummy for the changing market structure. Real per capita GDP is measured as the ratio between the real GDP (published by SARB) and the adult population.

To measure the importance of the tobacco-control policies for reducing tobacco consumption, a tobacco policy index is constructed for the period 1961–2016 based on the weights provided by Joossens and Raw (https://www.tobaccocontrolscale.org/TCS2010.pdf) for the different tobacco control policies. The index is constructed for policies other than cigarette taxes. Cigarette taxes are embedded in cigarettes prices and the individual effect of prices on consumption can be netted out in the demand model. The process of constructing the policy index follows a new tobaccocontrol scale (TCS) approach that measures the different non-pricing policies of countries^7,8^. The TCS, which quantifies the implementation of tobacco control policies at country level, is based on six policies described by the World Bank^25^.

The six policies are: 1) price increases through higher taxes on cigarettes and other tobacco products; 2) bans/restrictions on smoking in public and work places; 3) better consumer information, including public information campaigns, media coverage, and publicizing research findings; 4) comprehensive bans on the advertising and promotion of all tobacco products, logos and brand names; 5) large, direct health warning labels on cigarette packages and other tobacco products; and 6) treatment to help dependent smokers quit, including increased access to medications. In South Africa, the introduction of these policies started in the 1990s, allowing us to score them into a policy index using the TCS^8^. For the current study only five policies are scored, which excludes cigarette taxes (Table 1). In addition to excise taxes, South Africa has implemented a number of comprehensive tobacco-control policies in the Tobacco Product Control Act (TPCA) of 1993, including health warnings on cigarette packs and advertising material. This legislation was amended in 1999, 2001 and 2008 to include a ban on tobacco advertising, sponsorship, smoking in public and in work places and the sale of tobacco to minors^5,11^.

**Table 1:** The Tobacco Control Policies and Index in South Africa

*Year*	*The Tobacco Control Policies in South Africa from 1980*	*Scale*	*Cum. index*
**1980s**	Smoking prevalence peaked in the 1980s.	0	0
**1998/99**	Medical Research Council (MRC) publishes a paper showing that for every R1 of revenue, smoking costs government R5.	0	0
**1990/91**	The Minister of Health (MoH) is pushed into action and starts preparing the Tobacco Product Control (TPC) Bill.	0	0
**1992**	Taxes make up 30% of tobacco product prices.	0	0
**1993**	The MoH introduces the TPC Act of 1993, mandating that health warnings be added to cigarette packs and advertising material, and prohibiting smoking on public transport.	3	3
**1994**	The Minister of Finance (MoF) announced an increase in excise tax burden on cigarettes to 50% of the retail price over the number of years.	0	3
**1997**	To dissuade smokers, government raises taxes on tobacco products to 50% of cigarette retail prices.	0	3
**1999**	An amendment to the Tobacco Products Control Act bans tobacco advertising, the sale of tobacco to minors (age limit raised from 16 to 18 years) and increases regulations on smoking in public places, including the workplace. The MoH is awarded the WHO Tobacco Free World Award.	11	14
**2001**	The law banning public smoking comes into effect. Smokers may only smoke outside and in cordoned-off indoor areas. But restaurants can have smoking designated areas of up to 25% of the total area. Total ban on tobacco advertisement (enforced).	10	24
**2004**	Excise tax on tobacco products is raised to 52% of retail prices.	0	24
**2005**	South Africa ratifies the WHO’s Framework Convention on Tobacco Control (FCTC), which gives governments a framework for quickly passing and implementing evidence-based tobacco control laws.	0 24	0 24
**2008**	An amendment to the TPC Act aligns the country’s policies with FCTC guidelines by, for instance, raising the legal smoking age to 18 years, restricting tobacco sponsorship and promotion and mandating more extensive health warnings at points-of-sale.	2	26
**2012**	Draft regulations that would ban smoking in public places and certain outdoor public places, such as beaches and outdoor eating areas, are gazetted, but have not been passed into law.	0	26
**2013**	South Africa signs an international treaty to clamp down on the illegal trade in cigarettes.	0	26
**2016**	Minister of Health announces plans to introduce legislation that would: introduce plain packaging and pictorial health warnings; make indoor public places 100% smoke-free; ban vending machines; restrict pointof-sale marketing; regulate ENDS/ENNDS as tobacco products.	0	26

ENDS: electronic nicotine delivery systems. ENNDS: electronic non-nicotine delivery systems. Authors used The Tobacco Control Scale, 2010 (TCS) of Joossens and Raw. The index does not have a scale for restriction of the sale of tobacco to minors (age limit raised from 16 to 18 years as the policy is not included by Joossens and Raw).

Using these five policies, we develop an index using principal component analysis that sufficiently deals with the problems of multicollinearity and over-parameterisation as an overall indicator of the level of policy index. Principal component analysis has traditionally been used to reduce a large set of correlated variables into a smaller set of uncorrelated variables, known as principal components. This technique allows different measures of tobacco control policies to be expressed in terms of a single index. The complete table of the policies is given in Supplementary file Table S6.

A dummy variable is used to capture the change in market structure that occurred in 2010. The variable is coded 0 for periods before 2010, and 1 otherwise. Before 2010, British American Tobacco’s (BAT) main competitors were multinationals and other subsidiaries such as Philip Morris South Africa, Japan Tobacco International and Imperial Tobacco, but BAT was the unchallenged price leader. From 2010, there was a substantial change in the cigarette market structure in South Africa. The high profits earned by BAT and other multinationals attracted many small cigarette manufacturers and distributors, such as Gold Leaf Tobacco Company, Folha Manufacturers, and Savanna Tobacco Company SA among others, who undermined the established firms by selling at lower prices. During this period, there was also a substantial increase in illicit trade^11,26^.

The stationarity of the time series was tested using the Augmented Dickey-Fuller (ADF) and Phillip-Perron (PP) unit root tests^27,28^. The data were converted into logarithms in order to reduce spread (range) in their variability. Based on the ADF and PP tests, the hypothesis that the log of per capita consumption, the log of real prices and the log of real per capita GDP contain a unit root cannot be rejected at the 5% significance level. However, we fail to reject the assumption of stationarity after first differencing these variables. The critical values for the ADF and PP tests at first difference at 5% are -3.497 and -3.496, respectively. Compared to the test-statistic values, the variables are stationary at first differences (Figures S1 A and B; Table S1; Supplementary file), and thus, standard statistical inference is validated^29^. This suggests that a cointegration approach can be used to test for the existence of a long-run relationship between the variables.

### Co-integration and long-run equilibrium estimations

Before estimating the co-integrating vector, the appropriate lag length to be used in the estimations of the cointegration test and in the vector error correction model (VECM) was determined using the vector auto-regression (VAR) test. The lag length is selected if the majority of the selection criteria favor a particular lag^29^. The appropriate lag length used in the cointegration test and VECM model is presented in Supplementary file Table S2. The results show that the majority of the selection criteria, such as Akaike’s information criterion (AIC), the Hannan-Quinn Information Criteria (HQIC), and the Schwartz-Bayesian Information Criteria (SBIC), select the optimum lag length of 1 (Supplementary file, Table S2).

Cointegration was then tested using the Trace and the Maximum Eigenvalue tests^30^, which use a maximum likelihood procedure that jointly estimates the number of cointegration vectors to determine the existence of a long-run relationship between the variables. The results, presented in Supplementary file Table S3, show that there is one cointegrating vector among the variables. This signifies the existence of a long-run relationship among the variables that can be combined with the short-run dynamics using a Vector Error Correction Model^9,31^.

The long-run equilibrium model uses a doublelogarithmic demand equation, which gives a straightforward interpretation to the coefficients (elasticities). A conventional econometric model for estimating the demand for cigarettes is specified as follows:

lnQ_t_=α_0_+α_1_lnP_t_+α_2_lnY_t_+α_3_A_t_+α_4_D_t_+μ_t_

The variables *Q_t_, P_t_,Y_t_, A_t_* and *D_t_* have already been defined earlier, *α_i_* is the constant term where *i* = 1, 2, 3, 4 and *μ_t_* is the random error term. The vector error correction model (VECM) used to determine the long-run relationship is specified as^32^:

$$d\ln Q_{t}=\beta_{0}+\beta_{1}\sum_{i=1}^{j-1}d\ln Q_{t-i}+\beta_{2}\sum_{i=0}^{j-1}d\ln P_{t-i}+\beta_{3}\sum_{i=0}^{j-1}d\ln Y_{t-i}+\beta_{4}A_{t}+\beta_{5}D_{t}+\lambda_{t}ECT_{t-1}+\mu_{t}$$

where *j*-1 is the lag length, which is reduced by 1 since 1 lag is lost from differencing a VAR, *d* is the difference operator, *β_i_* represents the short-run dynamic coefficient of the model’s adjustment to longrun equilibrium, *λ_i_* is the speed of the adjustment parameter and *ECT_t-1_* is the error correction term, which is the lagged value of the residuals obtained from the cointegrating regression of the dependent variable on the regressors, and *μ_t_* is the stochastic error.

One concern that is often raised in the context of the estimation-of-demand equation is that cigarette prices are endogenous owing to the simultaneity of cigarette consumption and prices^33^. The market clearance price could be determined by the interaction between the demand and supply sides of the market and the estimates of price elasticity biased if the problem of endogeneity is ignored^4,33^. This study employs a two-stage least squares (2SLS) approach to address the potential endogeneity of cigarette prices. Excise taxes and lag of prices are the commonly used instruments for determining real cigarette prices^33^. The justification is that these two instruments serve the same purpose as price variable in affecting consumption behaviour, but are entirely independent of the individual’s smoking decision^33^. Moreover, the effectiveness of excise tax increases as a tool for reducing tobacco consumption and depends largely on how the tax increases impact the retail price11,34.

## RESULTS

The demand equation for annual cigarette consumption obtained from the VECM and 2SLS estimations is reported in Table 2. Supplementary file Table S4 presents the first stage estimates of the 2SLS method. The VECM was estimated at lag length of 1 with 1 cointegrating vector. Supplementary file Table S5 shows estimated results of the demand equation using specific dummies for the existing legislative Acts in South Africa. The relevant legislation is the Tobacco Product Control (TPC) Act 83 in 1993, that was amended in 1999, 2001 and 2008.

The estimated short-run dynamic coefficients of the real price and per capita income on per capita cigarette consumption are respectively -0.263 and 0.226 for the restricted model and -0.352 and 0.283 for the unrestricted model. The long-run price and income elasticities were estimated to be -0.722 and 0.394 for the restricted model, and -0.548 and 0.487 for the unrestricted model. The results suggest that price increases are an effective anti-smoking policy. A 10% increase in cigarette prices reduces per capita cigarette consumption by 5% to 7% in the long run. The positive and significant effect of income on cigarette consumption indicates that an increase in the income of smokers will result in higher levels of cigarette consumption, but to a lesser degree than the reduction caused by price increases.

**Table 2:** Results of the estimated VECM

*Variable*	*Short-run Dynamic*		*Long-run*		*2SLS*	
	*(1)*	*(2)*	*(3)*	*(4)*	*(5)*	*(6)*
Error correction term	-0.152***	-0.259**	-	-		
	(0.058)	(0.098)				
Log of cigarette prices	-0.263**	-0.352**	-0.722***	-0.548***	-0.523***	-0.589***
	(0.133)	(0.165)	(0.107)	(0.097)	(0.101)	(0.084)
Log of real per capita GDP	0.226*	0.283**	0.394***	0.487***	0.123***	0.052
	(0.203)	(0.217)	(0.077)	(0.052)	(0.044)	(0.040)
Policy index	-	-0.007	-	-0.016***	-0.011***	-0.006***
		(0.002)		(0.003)	(0.003)	(0.002)
Changing market structure	-	0.039	-	-0.146***	-0.042	-0.062
		(0.028)		(0.051)	(0.046)	(0.042)
Constant	0.017***	0.026	9.094	1.087	3.626***	5.008***
	(0.010)	(0.013)	(0.160)	(1.029)	(0.799)	(0.696)
**VECM diagnostic tests**						
Autocorrelation: LM	8.392 (0.495)					
Normality: Jarque-Bera	1.874 (0.931)					
Stability: Eigenvalue	4 unit moduli					
Durbin (score) χ2(1)					18.479***	3.223*
Wu-Hausman F(1,50)					24.625***	3.05*

Numbers in parenthesis are standard error.

***, **, * denote significance at 1%, 5% and 10%, respectively.

Columns (1) and (3) are the restricted models for the short- and longrun VECM, respectively. Columns (2) and (4) are the unrestricted models. Column (5) is the 2SLS model using real excise taxes as an instrument. Column (6) is the 2SLS model using lag of prices as an instrument.

The Durbin-Wu-Hausman test of exogeneity of regressors is used to test for the orthogonality of the unobserved disturbances in the demand equation. The test statistics in Table 2 suggest that cigarette prices are endogenous. Estimates of the first stage regression suggest that taxes and the lag of prices are valid instruments for cigarette prices. The strength of the instruments is tested using the robust F statistics (>10). This shows that the variables *tax* and *lagged prices* are strong predictors of cigarette prices. The 2SLS estimates show that a 10% increase in the price of cigarettes reduces cigarette consumption by 5.2% when cigarette excise taxes are used and 5.9% when the lag of prices is used.

The estimated coefficient of the policy index (-0.16) is highly significant in the long run, which suggests that the implementation of non-price tobacco control policies in South Africa reduces total cigarette consumption. The dummy for the change in market structure is negative and significant in the long run. Even though the change in market structure in 2010 (from a near monopoly to a more competitive market) led to lower prices being offered by new entrants to the market, the negative and significant coefficient indicates the percentage of the formal cigarette market (official cigarette consumption) that was lost to illicit trade post-2010.

The VECM uses stationary data at first differences and includes the lagged residuals of the long-run relationship as an explanatory variable. Coefficients from ECM represent the relationship in the short run, and the coefficient of the lagged residuals measures the speed of convergence to the long-run equilibrium. The error correction term is the speed of adjustment in the direction of long-term equilibrium after any deviation from the steady state. The error correction terms in Table 2 have the correct sign and are significant. This indicates that per-capita cigarette demand converges to steady state equilibrium at the speed of 15% in the restricted model (Table 2, column 1) and 26% in the unrestricted model (Table 2, column 2).

**Figure 2:**
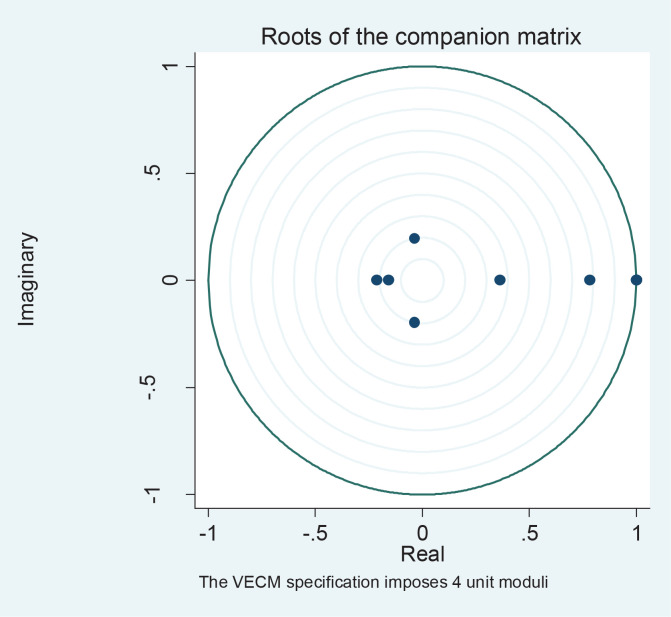
VECM stability test

In order to show that the demand equation was appropriate for the estimated results, some diagnostic tests were performed, namely: absence of serial correlation (Lagrange Multiplier test), normality of errors (Jarque-Bera test), and stability test. The results (Table 2) show the errors are normally distributed in the normal and there is absence of serial correlation. The stability of the VECM is confirmed using the graphs of the roots characteristics in Figure 2, as all the points fall within and on the unit root circle.

## DISCUSSION

According to other studies, the estimates of the price elasticity of demand for cigarettes in lowand-middle income countries are between -0.5 and -1.0, and between -0.25 and -0.5 for high-income countries^1^. The present estimate is within this range and lies between -0.52 and -0.57. The effect of income is reduced to 0.12 after controlling for endogeneity but remains significant (see Table 2 for 2SLS estimates).

It should be noted that the impacts of price and income in this study are lower than those in two studies conducted over the periods 1961 to 2004 and 1996 to 2000, respectively, in South Africa^5,6^. The differences in estimated value are most probably due to the differences in datasets and methodologies employed. In the first study, annual time series data from 1961 to 2004 obtained from different sources (Auditor-General, Statistics South Africa, Republic of South Africa and Tobacco Board) were used. Employing cointegration techniques, the price elasticity of demand for cigarettes was estimated at -0.78. Cigarette consumption was found to be highly responsive to income changes. However, no statistically significant relationship was found between tobacco advertising expenditure and cigarette consumption^5^. The second study used a quarterly dataset (which includes only wholesale cigarettes) obtained from a prominent South African manufacturer, while the present study uses annual retail sales data to estimate elasticities. It employs the unrestricted vector autoregression (VAR), which treats all variables as endogenous. This approach models each dependent variable as a function of its past values and the past values of other variables included in the model^6^. A recent study in South Africa using longitudinal data obtained from the South Africa National Income and Dynamic Study obtained negative price elasticity of demand for cigarettes, with significantly larger price elasticity estimates from the two-part model. The study found that price elasticity varies between -0.43 for economy brand cigarettes and 0.69 for the mid-price brands^13^. However, the study did not account for non-price tobacco control policies in the estimation of elasticities.

The policy index has a negative and statistically significant effect in the long run and for the 2SLS estimation. This suggests that introducing nonpricing tobacco-control legislation on tobacco use will effectively and significantly reduce cigarette demand in the long run. This is because, in the long run, smokers become aware of the regulations as they are implemented and enforced.

### Limitations

One of the major limitations of the study was that there has been a substantial increase in illicit cigarette trade in the recent years in South Africa. The estimated results do not account for illicit trade due to lack of credible information and data. Most of the illicit trade information in South Africa is produced by the tobacco industry, which might be providing misleading information. Therefore, the non-inclusion of an illicit trade variable in the model might lead to overestimated price elasticities. Another limitation remains the fact that the policy index is only calculated for the major tobacco control policies that are listed in Supplementary file Table S6. It may not be capturing minor tobacco control changes announced by the government over the years. More so, enforcements of tobacco control policies have often been a problem in South Africa. For example, although there is a ban on smoking in public places and public transport with minors, there is a disregard of their presence. In most of the public places and cities, nobody seems to mind the smoking of others. Therefore, there is a need to intensify public awareness campaigns and reenforce measures for the policies to be fully effective in reducing tobacco consumption.

## CONCLUSIONS

This study provides the combined effect of price and non-price policies on cigarette consumption in South Africa, a country that has a track record of effective tobacco control policies, yet experiences an increase in the burden of smoking-related disabilities. The results of this study show that the implementation and enforcement of antismoking policies would potentially reduce cigarette smoking, resulting in an improvement in public health. More than simple price increases are required to reduce cigarette consumption continuously in South Africa. As governments are committed to raising cigarette taxes as a way of reducing cigarette consumption, other non-price legislation should not be ignored.
