# Supplementary material for: The effects of price and non-price policies on cigarette consumption in South Africa
Source: Tob Induc Dis. 2020 Jul 23;18:62. doi: 10.18332/tid/123424 (PMC7398599; doi:10.18332/tid/123424)
Supplement: Supplementary file 1 [file TID-18-62-s1.pdf]

Supplementary file

Figure S1: Plot for variables at levels and first difference

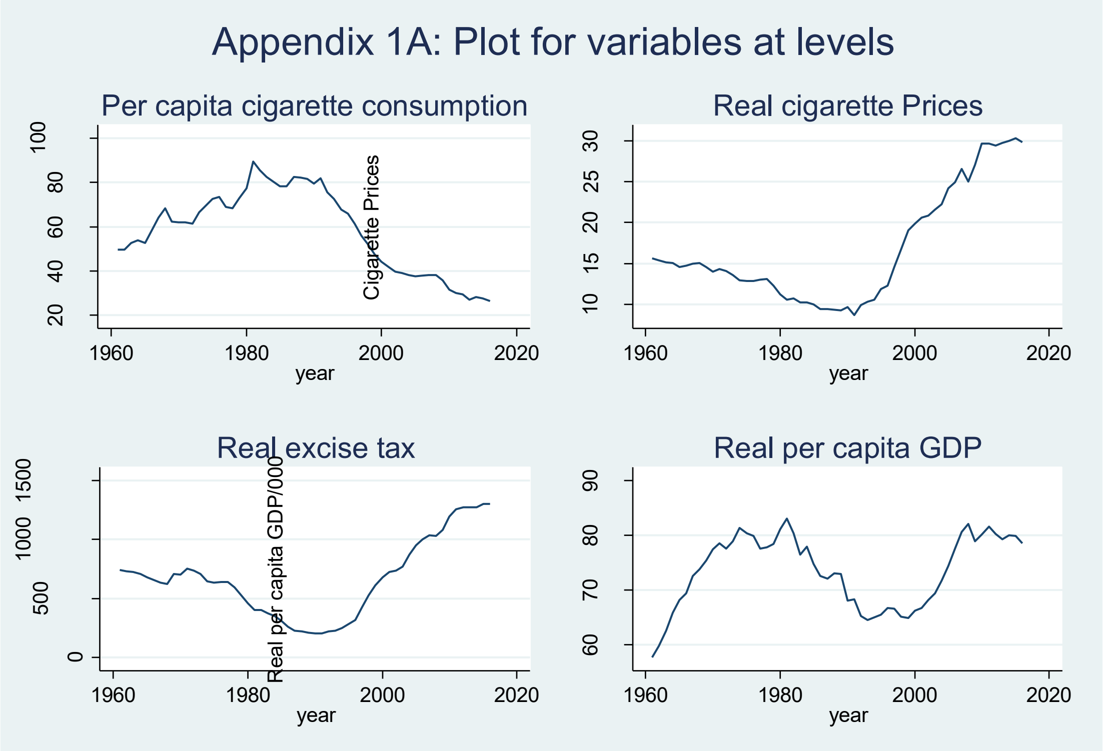

Source: By Authors

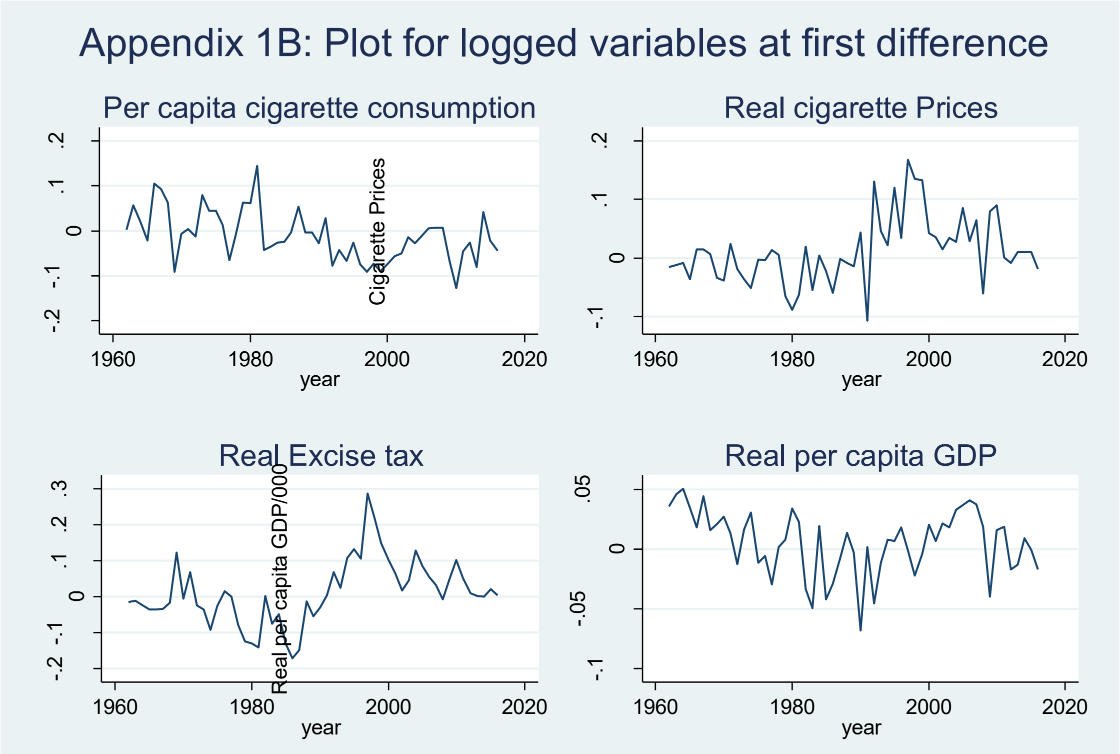

Source: By Authors

Table S1. Augmented Dickey-Fuller and Phillips-Perron Unit Root Tests

		Level		First difference	
**Variable**	**lags**	**ADF**	**PP**	**ADF**	**PP**
log(per capita consumption)	1	-1.698	-1.431	-4.474	-5.543
log(real prices)	1	-1.232	-1.115	-3.587	-5.522
log(real per capita gdp)	1	-3.127	-3.273	-4.175	-4.560

NB: 5% critical values are: -3.496 and -3.497 for the ADF test at level and first difference respectively; whereas the PP test critical values are -3.495 and -3.496 at level and first difference respectively.

Table S2. Optimal VAR Lag Selection criteria

lag	LR	Df	p	FPE	AIC	HQIC	SBIC
0				4.8e-06	-3.72732	-3.68417	-3.61475
1	416.27*	9	0.00	2.3e-09*	-11.3863*	-11.2137*	-10.936*
2	15.039	9	0.09	2.4e-09	-11.3294	-11.0273	-10.5414
3	7.2512	9	0.61	3.0e-09	-11.1227	-10.6911	-9.99696
4	13.705	9	0.13	3.3e-09	-11.0401	-10.479	-9.57665

Table S3. Johansen-Juselius Maximum Likelihood Test for Cointegration

		Trace Statistics		Maximum eigenvalue	
**Null**	**Eigenvalue**	**statistic**	**5% critical value**	**statistic**	**95% critical value**
r=0	N/A	55.1446	29.68	47.6385	20.97
r<1	0.57943	7.5061*	15.41	7.3605	14.07
r<2	0.12526	0.1456	3.76	0.1456	3.76

Table S4. First Stage Estimates of 2SLS

	**Excise taxes**	**Lagged prices**
log of real excise taxes	0.384***	-
	(0.0368)	
log of lagged prices	--	1.0257***
		(0.0674)
log of real per capita gdp	0.0224	0.0600*
	(0.0436)	(0.0339)
policy index	0.009***	.007*
	(0.0017)	(0.0018)
changing market structure	-0.0755*	-.0411
	(0.0429)	(.0325)
Constant	-0.1506	-0.874
	(0.7734)	(0.5944)
**Number of obs**	56	55
F(4, 51)	391.46	699.38
R-squared	0.9685	0.9824
Adj R-squared	0.9660	0.9810

Numbers in parenthesis are standard error; ***, **, * denote significance at 1%, 5% and 10% respectively.

Table S5. Regression results using specific dummies

	(1)	(2)	(3)
log of cigarette prices	*-0.447****	*-0.447****	*-0.566****
	(0.083)	*(0.083)*	*(0.076)*
log of real per capita gdp	*0.182****	*0.182****	*0.117****
	(0.044)	*(0.044)*	*(0.043)*
changing market structure	*-0.234****	*-0.234****	*-0.211****
	(0.045)	*(0.045)*	*(0.043)*
Legislation 1	*-0.224****	*-0.224****	*-0.174****
	(0.043)	*(0.043)*	*(0.041)*
Legislation 2	*-0.465****	*-0.465****	*-0.336****
	(0.089)	*(0.089)*	*(0.084)*
Legislation 3	*-0.486****	*-0.486****	*-0.322****
	(0.114)	*(0.114)*	*(0.108)*
Constant	*2.908****	*2.908****	*4.089****
	(0.760)	*(0.760)*	*(0.730)*
Observations	*56*	*56*	*55*
R-squared	*0.970*	*0.970*	*0.973*
Durbin (score) chi2(1)		*9.587 (p = 0.002)*	*0.016 (p = 0.898)*
Wu-Hausman F(1,48)		*9.916 (p = 0.003)*	*0.014 (p = 0.907)*

Numbers in parenthesis are standard error; ***, **, * denote significance at 1%, 5% and 10% respectively.

Note:

Columns (1) is the long run VECM results using specific dummies
Column (2) is the 2SLS model using real excise taxes as an instrument
Column (3) is the 2SLS model using lag of prices as an instrument

Specific dummies have been used for the existing tobacco legislation in South Africa.
Tobacco Products Control Act, Act no. 83 of 1993
Was amended by:
Tobacco Products Control Amendment Act, no12 of 1999;
Tobacco Products Control Amendment Act, no 23 of 2001 &
Tobacco Products Control Amendment Act, no 63 of 2008.

Table S6. Tobacco Control Scale by Joossens and Raw (2011)

No	Tobacco Control Policy	Scale
**1**	**Price of cigarettes and other tobacco products**	**30**
**2**	**Smoke free work and other public places**	**22**
	**Workplaces excluding cafes and restaurants - only one of**	10
	Complete ban without exceptions (no smoking rooms); enforced	10
	Complete ban, but with closed, ventilated, designated smoking rooms under very strict rules; enforced	8
	Complete ban, but with ventilated, designated smoking rooms (not areas or places); enforced	6
	Meaningful restrictions; enforced (75% of the workplace are smoke free)	4
	Legislation, but not enforced	2

	**Cafes and restaurants - one only of**	8
	Complete ban; enforced	8
	Complete ban, but with closed, ventilated, designated smoking rooms (not areas or places); enforced	6
	Meaningful restrictions; enforced (50% of bars and restaurants are smoke free)	4
	Legislation, but not enforced	2
	**Public transport and other public places - additive**	4
	Complete ban in train without exceptions	1
	Complete ban in other public transport without exceptions	1
	Complete ban in educational, health, government and cultural places without exceptions OR	2
	Ban in educational, health, government and cultural places, but with designated smoking areas or rooms	1
**3**	**Spending on public information campaigns**	**15**
**4**	**Comprehensive bans on advertising and promotion**	**13**
	Points for each type of ban included - additive	1
	Complete ban on tobacco advertising on television and radio	2
	Complete ban on outdoor advertising (e.g posters)	2
	Complete ban on advertising in print media (e.g newspapers and magazines)	1.5
	Complete ban on indirect advertising (e.g cigarette branded cloths, watches …)	1
	Ban on display of tobacco products at the point of sales	2
	Ban on point of sale advertising	1
	Ban on cinema advertising	1
	Ban on sponsorship	1
	Ban on internet advertising	0.5
**5**	**Large direct health warning labels**	**10**
	Plain packaging (the removal of trademarks, logos, colours and graphics, except for the government health warning and for the brand name, presented in a standardized typeface) in combination with pictorial health warnings in the front and the back of the tobacco product packages	4
	Size of warning - one only of	3
	50% or less of packet	1
	51 - 79% of packet	2
	80% or more of packet	3
	Pictorial health warnings - additive	3
	Pictorial health warnings on cigarette packs	2
	Pictorial health warning on hand rolling tobacco	1
**6**	**Treatment to help dependent smokers stop**	**10**
	Recording of smoking status in medical notes	1
	Legal or financial incentive or record to record smoking status in all medical notes or patient files	1
	Brief advice in primary care	1
	Family doctors reimbursed for providing brief advice	1
	Quit line	2
	National quit line or quit line in all major regions of country	1
	Quit line counsellors answering at least 30 hours a week (not recorded)	1
	**Network of smoking cessation support and its reimbursement - one only of**	**4**
	Cessation support network covering whole country, free	4
	Cessation support network but only in selected areas, eg major cities; free	3
	Cessation support network covering whole country, partially or not free	3
	Cessation support network but only in selected areas, eg major cities; partially or not free	2
	**Reimbursement of medications - one only of**	**2**
	Medications totally reimbursed or free to users OR	2
	Medications partially reimbursed	1

Source: Joossens and Raw (2011)

© 2020 Tingum E.N. et al.
